# Introduction of radiation therapist‐led adaptive treatments on a 1.5 T MR‐Linac

**DOI:** 10.1002/jmrs.643

**Published:** 2022-12-26

**Authors:** Maddison Picton, David Crawford, Michael Jameson, Stacy Alvares, Louise Hogan, Conrad Loo, Zoe Moutrie, Urszula Jelen, Claire Pagulayan, Nicolle Dunkerley, Tania Twentyman, Jeremy de Leon, Vikneswary Batumalai

**Affiliations:** ^1^ GenesisCare Alexandria New South Wales Australia; ^2^ School of Clinical Medicine, Faculty of Medicine and Health UNSW Sydney Kensington New South Wales Australia; ^3^ Department of Radiation Oncology South West Sydney Local Health District Sydney New South Wales Australia

## Abstract

The introduction of magnetic resonance (MR) linear accelerators (MR‐Linac) marks the beginning of a new era in radiotherapy. MR‐Linac systems are currently being operated by teams of radiation therapists (RTs), radiation oncology medical physicists (ROMPs) and radiation oncologists (ROs) due to the diverse and complex tasks required to deliver treatment. This is resource‐intensive and logistically challenging. RT‐led service delivery at the treatment console is paramount to simplify the process and make the best use of this technology for suitable patients with commonly treated anatomical sites. This article will discuss the experiences of our department in developing and implementing an RT‐led workflow on the 1.5 T MR‐Linac.

## Graphical abstract

This work provided an overview of processes involved in the development of an RT‐led workflow for the adapt‐to‐position (ATP) approach on the 1.5 T MR‐Linac.

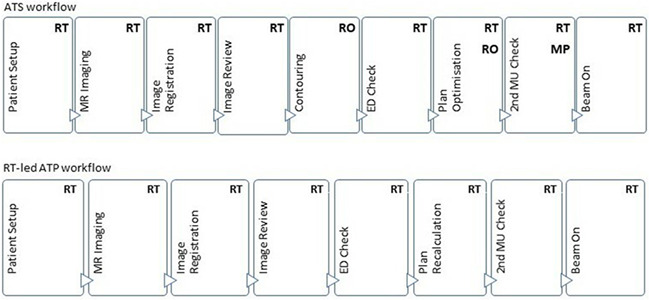

## Introduction

Magnetic resonance (MR) imaging has been shown to provide valuable additional information for many tumour sites and associated normal tissues due to its excellent soft tissue discrimination and functional information.^1^ Hybrid technologies combining linear accelerators (linacs) and MR scanners (MR‐Linacs) mark the beginning of a new era. MR‐guided adaptive radiotherapy (MRgART) enables daily adaptive radiotherapy allowing the treatment plan to be personalised to the patient's anatomy at the time of treatment.^2^ This ensures a precise localisation and real‐time tracking of the tumour^3^ and organs at risk (OAR), allowing further potential for dose escalation.

The traditional radiotherapy treatment planning process may span between days to weeks with the exception of ‘plan and treat’ cases where the planning workflow may be reduced to hours. By contrast, the process of MRgART condenses the planning workflow into minutes at the treatment console. As a result, the process requires input from the multidisciplinary radiation oncology professionals; radiation therapists (RTs), radiation oncology medical physicists (ROMPs) and radiation oncologists (ROs) at the treatment console. This is resource‐intensive and logistically challenging,^4^ especially for services that span across multiple locations. If MRgART can be delivered similar to standard image‐guided radiotherapy (IGRT) involving traditional RT‐led service at the treatment console, these processes can be simplified and become more efficient.

This work aimed at providing an overview of processes involved in the development of an RT‐led approach to MRgART on the 1.5 T MR‐Linac.

## Overview

The concept of MRgART allows treatment plan adaptation for every fraction with options of multiple adaptation methods. There are two workflows available when treating on the Unity MR‐Linac (Elekta AB, Stockholm, Sweden): adapt‐to‐shape (ATS) and adapt‐to‐position (ATP) using Monaco treatment planning system (Elekta AB). The ATS^5^ is a complex approach that involves a full plan adaptation with contour propagation and (re)contouring and plan approval completed by the treating radiation oncologist. By contrast, the ATP^5^ approach involves a less complex virtual plan isocentre shift, which only requires adapting the multi‐leaf collimator (MLC) leaves according to translational corrections with no further intervention to the contours. In our centre, ‘optimise shapes’ adaptation method is used for ATP where the default optimisation parameters are applied to change the segment weights and allows the optimiser to adjust the MLC leaf positions of the adapted plan to better match the reference plan dose distribution. This provides an opportunity for the plan to meet target and OAR dose without the full adaptive workflow involved in ATS. Therefore, ATP was adopted for the RT‐led workflow.

## Development

**Figure 1:**
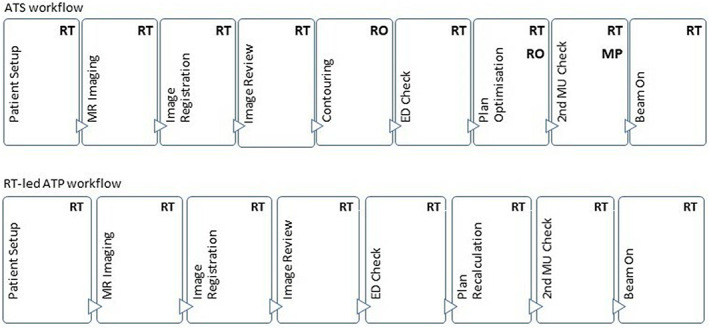
Roles of each of the craft groups for ATS and RT‐led ATP workflows. RT, radiation therapist; RO, radiation oncologist; MP, medical physicist; ED, electron density; MU, monitor unit.

Multidisciplinary team meetings involving RTs, ROs and ROMPs were held to identify the appropriate tumour site and cases suitable for the RT‐led workflow. It was decided that intact prostate cancer patients receiving more than five fractions would benefit from the RT‐led workflow, as prostate cancer is the most common clinical indication for treatment on MR‐Linac in our centre.^6^ Figure 1 shows the roles of each of the craft groups comparing ATS and RT‐led ATP workflow. The development process was divided into three main components: framework, tolerance and action levels, and staff credentialing.

### Framework

#### ATS workflow

**Figure 2:**
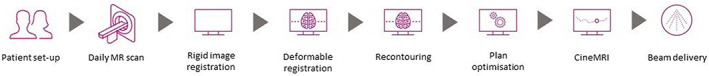
Adapt‐to‐shape workflow (Image courtesy of Elekta).

The ATS workflow is used for the first five fractions of every patient. This ultimately creates a library of patient‐specific data sets and plans that can be utilised for the RT‐led ATP workflow. The steps involved in the ATS workflow are highlighted in Figure 2 and briefly described here. The patient is positioned on the MR‐Linac according to the simulation instructions, and a T2‐weighted MR scan is obtained. For the first fraction, the planning computed tomography (CT) is registered to the online MR scan, and using a combination of rigid and deformable image registration methods, the contours from the reference plan are propagated onto the online MR image. From fraction 2 onwards, the online MR scans are registered to a previous MR scan with visually similar OAR sizes and characteristics to enable superior image fusion and deformable registration. The selection of the most suitable previous MR scan is based on the ‘Unity handover document’ where volumes and doses delivered to all structures are recorded on a daily basis. The document serves as a tool to support decision‐making for subsequent fractions. Once the contours are propagated, the RO may edit any contours where needed, based on the anatomy of the day. Following this, the treatment plan is re‐optimised using ‘optimise shapes’ adaptation method and approved by the RO. The motion monitoring 2D cineMR images are reviewed prior to beam‐on and display the planning target volume (PTV) location relative to the patient's anatomy. Treatment is initiated while monitoring the PTV using 2D cineMRI.^7^

#### RT‐led ATP workflow

**Figure 3:**
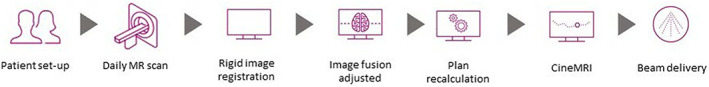
RT‐led adapt‐to‐position workflow (Image courtesy of Elekta).

Once the library of datasets and plans has been created from the first five fractions, the RT‐led ATP workflow can be used from fraction 6 without the RO present. The steps involved in the RT‐led workflow are displayed in Figure 3 and briefly described here. Following patient set up, a T2‐weighted MR scan is obtained. A previous MR image will be selected from the library of plans based on the Unity handover document and a visual qualitative comparison made by the RT of the bladder and rectum size to be fused and adapted to. The RT performs an automatic fusion and contour propagation, where the PTV, rectum and bladder volumes are assessed in Monaco. The MR fusion is adjusted to provide a superior match of the volumes for that fraction. The bladder and rectum volume is also assessed ensuring the contours visually cover the anatomy. Finally, the patient contour is reviewed by at least two credentialed RTs, ensuring that the variation is less than 5 mm in any direction, for regions within 2 cm superior and inferior to maximum extent of the PTV. If all metrics are met and the volumes are within tolerance, the plan is adapted using the ATP ‘optimise shapes’ method and treatment delivery proceeds. In the event of dose constraint violations for any of the structures, the RO is called to provide the ATS workflow for plan review and approval.

### Tolerance and action levels

**Figure 4:**
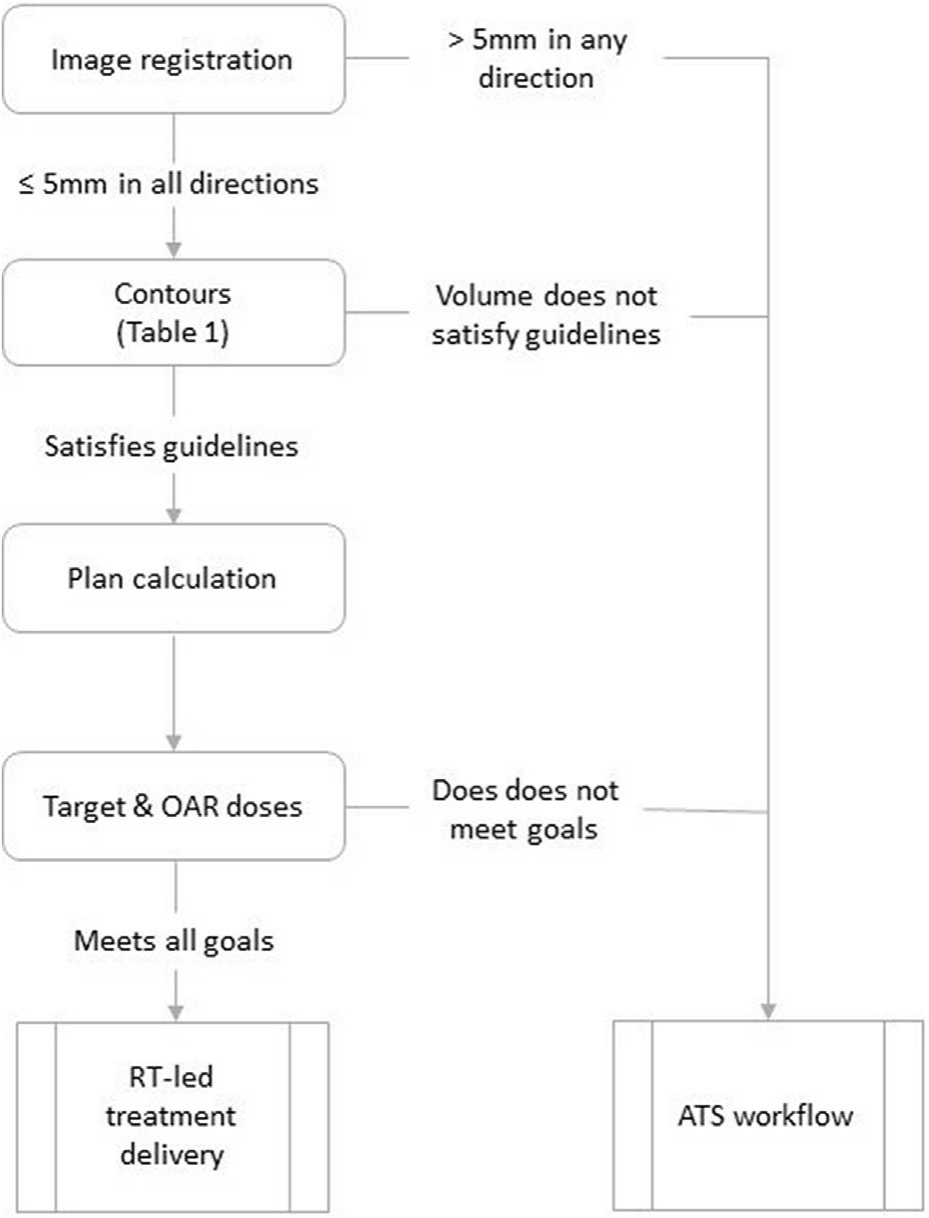
Tolerance and action level for RT‐led workflow.

Respiratory and organ motion contribute to geometrical uncertainty in radiotherapy, and longer time on the radiotherapy couch may be associated with larger uncertainties. Therefore, due to the adaptive nature of each fraction, it is essential that critical online decisions are made swiftly and consistently. This required close multidisciplinary teamwork to define and optimise treatment workflows and thresholds for decision‐making and action levels. Strict tolerance and action levels were developed for the RT‐led workflow (Table 1). A decision tree (Fig. 4) was established to determine whether an RT‐led workflow was acceptable for that fraction. If a patient met all of the guidelines and was treated using the RT‐led ATP workflow, the treating RTs will document the fraction as ‘RT‐led treatment session’ in the Unity handover document. This record serves as a clinical handover between RTs and provides evidence for future audit and process improvement.

**Table 1:** Tolerance level for RT‐led adapt‐to‐position workflow.

	Tolerance
Clinical target volume	Prostate matched within propagated clinical target volume (≤5 mm in all directions)
Rectum	Rectum anatomy matched within propagated rectum contour (≤5 mm in all directions)
Bladder	Bladder anatomy matched within propagated bladder contour (≤2 cm in all directions)

### Credentialing

A credentialing framework was developed for this workflow using the experience of another Elekta Unity site (The Royal Marsden) through personal communication, as well as the input from internal ROs and RTs. This credentialing programme enabled those RTs working on the MR‐Linac to be trained and competent in the RT‐led workflow in an online setting. The credentialing consists of both offline and online components to be completed by RTs. In the offline component, the RTs are required to prepare 20 MRgART plans using the RT‐led workflow; then, these are reviewed by a qualified RO. Once these 20 plans are signed off and deemed clinically acceptable, the RTs are required to complete 10 plans online using the RT‐led workflow under the supervision of the prescribing RO. The credentialing framework covers appropriate reference plan choice, image registration, identification of the anterior wall of rectum, bladder volume assessment and the understanding of the decision tree and when to engage the RO.

## Discussion

The RT‐led adaptive workflow for prostate cancer using MR‐Linac has been successfully implemented at St Vincent's Hospital, Sydney, GenesisCare. Since the initiation of this workflow, eight RTs have been successfully credentialed. The RT‐led workflow has broadened the responsibilities of RTs and allowed for role expansions since the implementation of this approach. We found that the implementation of this workflow has reduced the overall treatment times by 10 to 15 min and relieved ROs from being at the console for all fractions. The time saved could be attributed to the seamless workflow performed by RTs alone as opposed to having multiple work groups performing various tasks.

Online tasks required as part of MRgART increase the workload of key radiotherapy staff. With an increasing number of patients being treated on MR‐Linac systems, the transition of tasks from ROs to RTs is being widely investigated. A recent study reported that the general consensus from focus group interviews of RTs, ROs and ROMPs was to move towards RT‐led workflows.^8^ Another study reported results of a physician‐free workflow for the MRIdian MR‐Linac, concluding that the process had a success rate of 97.5% where plans were correctly adapted to the gold standard.^9^ Similarly, another study reported that early evaluation of the RT‐led framework after treatment of 10 patients has required minimal online clinician input (1.5% of 200 fractions delivered).^10^ More recently, the investigation of RT‐led daily online contouring for prostate cancer treatment reported that the contours were acceptable for clinical use in 94.2% fractions.^11^ These findings suggest that the RT‐led workflow is feasible and can be implemented into a routine clinical setting. However, comprehensive training and credentialing is required for a safe and successful RT‐led treatment.^10^, ^12^

We anticipate the current working model of MR‐Linac systems will continue to evolve as RT‐led treatment becomes more prevalent in routine practice and new anatomical sites or clinical indications are introduced. Artificial intelligence may also be integrated into this process as it becomes applicable to the workflow. Moving forward, roles and responsibilities may cross traditional boundaries, to enable a prompt and less resource‐intensive MRgART.

## Conclusion

The implementation of an RT‐led workflow for prostate cancer using MR‐Linac has presented a number of benefits including efficiency gained through a seamless treatment delivery process, broadening RTs' skillsets and role expansions. The development of the RT‐led workflow required close collaboration between RTs, ROMPs and ROs. This workflow has provided a framework to expand the process to other tumour sites.

## Conflict of interest

The author declares no conflict of interest.
